# Phylogenomics, plastome degradation and mycoheterotrophy evolution of Neottieae (Orchidaceae), with emphasis on the systematic position and Loess Plateau-Changbai Mountains disjunction of *Diplandrorchis*

**DOI:** 10.1186/s12870-022-03906-0

**Published:** 2022-11-01

**Authors:** Huan-Wen Peng, Lian Lian, Jun Zhang, Andrey S. Erst, Wei Wang

**Affiliations:** 1 State Key Laboratory of Systematic and Evolutionary Botany, Institute of Botany, Chinese Academy of Sciences, Beijing, 100093 China; 2 University of Chinese Academy of Sciences, Beijing, 100049 China; 3 Forestry College, Beihua University, Jilin, 132013 China; 4 Central Siberian Botanical Garden, Russian Academy of Sciences, Zolotodolinskaya str. 101, Novosibirsk, 630090 Russia

**Keywords:** Biogeography, Loess Plateau-Changbai Mountains disjunction, Mycoheterotrophy, Orchidaceae, Phylogenomics, Plastome degradation

## Abstract

**Background:**

Mycoheterotrophy is a unique survival strategy adapted to dense forests and has attracted biologists’ attention for centuries. However, its evolutionary origin and related plastome degradation are poorly understood. The tribe Neottieae contains various nutrition types, i.e., autotrophy, mixotrophy, and mycoheterotrophy. Here, we present a comprehensive phylogenetic analysis of the tribe based on plastome and nuclear ITS data. We inferred the evolutionary shift of nutrition types, constructed the patterns of plastome degradation, and estimated divergence times and ancestral ranges. We also used an integration of molecular dating and ecological niche modeling methods to investigate the disjunction between the Loess Plateau and Changbai Mountains in *Diplandrorchis*, a mycoheterotrophic genus endemic to China that was included in a molecular phylogenetic study for the first time.

**Results:**

*Diplandrorchis* was imbedded within *Neottia* and formed a clade with four mycoheterotrophic species. Autotrophy is the ancestral state in Neottieae, mixotrophy independently originated at least five times, and three shifts from mixotrophy to mycoheterotrophy independently occurred. The five mixotrophic lineages possess all plastid genes or lost partial/all *ndh* genes, whereas each of the three mycoheterotroph lineages has a highly reduced plastome: one lost part of its *ndh* genes and a few photosynthesis-related genes, and the other two lost almost all *ndh*, photosynthesis-related, *rpo*, and *atp* genes. These three mycoheterotrophic lineages originated at about 26.40 Ma, 25.84 Ma, and 9.22 Ma, respectively. *Diplandrorchis* had presumably a wide range in the Pliocene and migrated southward in the Pleistocene.

**Conclusions:**

The Pleistocene climatic fluctuations and the resultant migration resulted in the Loess Plateau-Changbai Mountains disjunction of *Diplandrorchis*. In the evolution of mycoheterotrophic lineages, the loss of plastid-encoded genes and plastome degradation are staged and irreversible, constraining mycoheterotrophs to inhabit understories with low light levels. Accordingly, the rise of local forests might have promoted the origin of conditions in which mycoheterotrophy is advantageous.

**Supplementary Information:**

The online version contains supplementary material available at 10.1186/s12870-022-03906-0.

## Background

Mycoheterotrophy is a survival strategy for adult organismal adaptation to forests with dense overstories and low light levels, in which carbon is obtained fully from symbiotical mycorrhizal fungi [1–3]. This unique nutrition type has attracted biologists’ attention for centuries [3–5]. More than 450 angiosperm species, which are distributed in ten families, possess of mycoheterotrophic habit [6]. Because of their sensitivity to environments and usually small population sizes, many mycoheterotrophic species are at risk of extinction due to climate change and anthropogenic activities [7]. However, the evolutionary origin of mycoheterotrophic habit, especially related with possible environmental changes, is still poorly understood.

Compared to mycoheterotrophy, autotrophy is more common nutrition type, in which adult organisms obtain carbon from inorganic sources by photosynthesis [5]. Mycoheterotrophy is derived from autotrophy and regarded as an extreme end of mycorrhizal symbiosis [6]. Between autotrophy and mycoheterotrophy, there is an intermediate type, mixotrophy, in which adult organisms use both organic and mineral carbon resources [8]. To date, it is unknown whether the evolutionary shift from autotrophy to mycoheterotrophy can occur without mixotrophy.

In land plants, generally the plastid genomes (plastomes) are highly conserved in structural organization and gene content [9, 10]. Nevertheless, mycoheterotrophic plants lack photosynthetic capacity, and correspondingly photosynthesis-related genes and some housekeeping genes are lost, resulting in a highly reduced plastome [11–13]. The loss of plastid-encoded genes is usually staged and irreversible [14]. The NADH dehydrogenase-like genes (*ndh*) are lost first, followed by most photosynthesis-related genes (i.e., *psa*, *psb*, *pet*). Rubisco large subunit (*rbcL*), adenosine triphosphate synthase (*atp*), and plastid-encoded RNA polymerase genes (*rpo*) are kept in the early stage of photosynthesis loss, and housekeeping genes involved in plastid translation and other specific functions are lost finally. This model of sequential plastome degradation associated with mycoheterotrophy remains debated [13]. Moreover, different mycoheterotrophic lineages may display distinct gene loss patterns [15–17]. To better understand the relationship between plastome degradation and shift of nutrition types, we need to study more taxa and investigate gene loss patterns in a phylogenetic context.

The tribe Neottieae (Epidendroideae, Orchidaceae), consisting of six to seven genera with ca. 180 species, is primarily restricted to the temperate and subtropical forests in the Northern Hemisphere with a few species extending into tropical alpine and montane regions [18, 19]. This tribe has various nutrition types: autotrophic type occurs in *Palmorchis* and some species of *Cephalanthera*, *Epipactis* and *Neottia*; mixotrophic type occurs in *Limodorum* and some species of *Cephalanthera*, *Epipactis* and *Neottia*; and mycoheterotrophic type occurs in *Aphyllorchis*, *Diplandrorchis*, and some *Cephalanthera* and *Neottia* species [17, 18]. Thus, Neottieae offers a remarkable opportunity to unravel the effect of shift of nutrition types on plastome degradation. A few studies have been devoted to investigate plastome degradation of Neottieae and have indicated that mycoheterotrophic habit is distributed in three clades within this tribe [17, 20, 21]. However, the evolutionary patterns of the nutrition types and plastid gene loss have not been inferred in a phylogenetic framework. Moreover, the potential environmental factors driving the origins of mycoheterotrophs in Neottieae have never been explored.

In Neottieae, only the monotypic *Diplandrorchis* has not been sampled in any molecular study. This genus was first described by Chen [22] based on the collection *S. C. Chui et J. C. Chu 245* in Huanren County of Liaoning Province in northeastern China, Mt. Changbai, and is unique in that green leaves and rostellum are absent, and in having actinomorphic flowers, three similar sepals, three similar petals without a modified lip, and two fertile stamens (one being opposite to the dorsal sepal and another to the median petal). Govaerts [23] informally reduced it to *Neottia*, but did not give any reason. Chen et al. [18] still considered it to be a distinct genus. For a long time, *D. sinica* was only found in its type locality and was listed as a “critically endangered” species [7]. Recently, two small populations of this species were reported from the Loess Plateau in Huangling County of Shaanxi Province and Ning County of Gansu Province [24]. This means that *D. sinica* displays an interesting disjunction between the Loess Plateau and Changbai Mountains.

Here, we first build a phylogenetic tree for Neottieae using 79 plastid protein-coding and one nuclear ITS DNA regions. Within the phylogenetic framework, we aim to (1) clarify the systematic position of *Diplandrorchis*, (2) infer the relationship between plastome degradation and shift of nutrition types in Neottieae, and (3) explore potential environmental factors driving the evolutionary origins of mycoheterotrophs. We also discuss the possible causes for the formation of the Loess Plateau-Changbai Mountains disjunct distribution.

## Materials and methods

### Taxon sampling and data assembly

We sampled 29 species, representing all seven of the currently recognized genera of Neottieae (Table S1). Our sampling covers the various nutrition types of each genus in this tribe. Two individuals of *D. sinica* were sampled from two geographic areas, the Loess Plateau, in Gansu Province, and the Changbai Mountains, in Jilin Province, respectively. Based on Serna-Sánchez et al. [25], *Sobralia callosa* of Sobralieae was selected as the outgroup. The sample of *D. sinica* from Gansu Province was newly collected with the permission from local Forest Department for this study and was deposited in Herbarium, Institute of Botany, the Chinese Academy of Sciences, Beijing (PE). Huan-Wen Peng and Jun Zhang performed formal identification of the sample after collection.

Total genomic DNA was extracted from silica-dried tissue materials using a modified CTAB method [26]. DNA library construction and paired-end Illumina sequencing were done by Novogene Bioinformatics Technology Company Limited (Beijing, China), which generated ~ 10 Gb of 150-bp paired-end reads. The plastome was de novo assembled using GetOrganelle pipeline [27] and was annotated using GeSeq [28] with the plastome of *Neottia fugongensis* (NC_030711) as the reference. We used Geneious v.8.0.4 [29] to correct the annotation and OGDRAW v.1.3.1 [30] to visualize the circular plastome map. Pseudogenes were identified following the method of Lallemand et al. [17]. All 79 plastid protein-coding gene regions were extracted, each of which was aligned using MAFFT v.7 [31] with default settings, followed by manual adjustment. The concatenated plastid supermatrix comprised 69,137 aligned nucleotides. We also assembled an ITS dataset of 664 aligned nucleotides with the same taxa.

### Phylogenetic analyses and divergence time estimation

We first used the maximum likelihood (ML) method to perform nonparametric bootstrap analyses for the ITS and plastome datasets in RAxML v.7.0.4 [32]. No significant bootstrap support for conflicting nodes was evident between these two datasets (here considered exceeding 70%), and the ITS and plastome datasets were therefore combined for subsequent analyses. Phylogenetic analyses for the combined dataset were carried out using ML and Bayesian methods. RAxML was performed with the GTR + G nucleotide substitution model for ITS and plastome regions, and the fast bootstrap option, using 1000 replicates.

Bayesian analysis was conducted in BEAST v.2.3.0 [33], which can co-estimate topology, substitution rates and node ages. We used two secondary calibration points with a normal prior distribution, taking ages estimated in the recent broader study of Orchidaceae [25]. The root age was constrained to 60.49 Ma (standard deviation [SD] = 5.0), which is the stem group age of Neottieae. The crown group age of Neottieae was constrained to 56.26 Ma (SD = 6.0). Using the uncorrelated lognormal relaxed clock model, birth-death tree prior, and the GTR + G model for two independent partitions (ITS vs. plastome), we ran the analysis for 50,000,000 Markov chain Monte Carlo (MCMC) generations sampling every 5000 generations. Tracer v.1.7.1 [34] was used to check chain convergence and the adequate effective sample size values (> 200). After discarding the first 25% of samples as burn-in, the maximum clade credibility (MCC) tree with posterior probability (PP), mean age and 95% highest posterior density (HPD) for each node was generated using TreeAnnotator v.2.3.0 [33].

### Evolutionary inference of nutrition types

Evolutionary inference of nutrition types for Neottieae was performed in BayesTraits v.3.0.1 [35], as implemented in RASP v.4.2 [36]. Nutrition types were categorized into three states: autotrophy, mixotrophy, and mycoheterotrophy. The data were obtained from the literature [2, 5, 18, 37]. We randomly sampled 1000 trees from the phylogenetic inference in BEAST (excluding burn-in). The MCMC chain of BayesTraits was run for 5,000,000 generations, discarding the results sampled during the first 500,000 generations as burn-in.

### Ancestral range estimation

Based on the floristic characteristics of Takhtajan [38] and distributions of Neottieae (Table S1; [39]), we defined five bioregions: Southeast Asia (including India and southern China), temperate Eurasia (including northern Africa), Africa, North America, and Neotropics. The maximum range size was restricted to three because no sampled species occurs in more than three biogeographic regions. The validity of the *j* parameter describing jump-dispersal or founder-event speciation remains controversial [40, 41], but this parameter is informative for modeling the biogeography of organisms with strong dispersal abilities [42]. Considering that the seeds of orchids are dust-like and wind-dispersed [18, 43], we tested the three models (DEC, DIVA-like and BayArea-like) including or not the *j* parameter. The DEC model (with the highest AICc_wt) outperformed the other five models (Table S2). To account for phylogenetic uncertainties, ancestral range estimation was conducted using BioGeoBEARS [44] under the statistical DEC model, as implemented in RASP v.4.2 [36]. The MCC tree and 1000 posterior trees subsampled from BEAST analysis were imported as input files. Referring to Givnish et al. [45], we specified dispersal probabilities between pairs of areas for three separate time slices (Table S3).

### Species distribution modeling

We used MaxEnt v.3.3.3 [46] to model historic and current potential distributions for *D. sinica*. MaxEnt uses the maximum entropy model to estimate potential niches of species and predict habitat suitability. It has been shown to have better predictive power than other methods, even though only a small number of localities are obtained [47–49]. Seven georeferenced records of *D. sinica* were collected from the literature, herbaria, and our field survey (Table S4). “Pseudo-occurrences” need to be added to reach the minimum number required for ecological niche modeling (ENM) [49, 50]. Here, we added four pseudo-occurrences for each record by locating 1 km to the east, west, north and south based on Li et al. [50]. After removing duplicate (pseudo-)occurrences within a buffer of < 1 km, we retained 31 records. Considering the origination and diversification ages of *D. sinica* (see below Results), 14 bioclimatic variables were downloaded from PaleoClim [51] in four periods: mPWP (mid-Pliocene Warm Period; 3.205 Ma), MIS19 (Marine Isotope Stage 19; ~ 0.787 Ma), Last Interglacial (~ 0.13 Ma), and current (1979–2013). After removing the variables whose correlation coefficient was > 0.85, the resultant five variables were used to predict the potential distribution of *D. sinica*: temperature seasonality, mean temperature of coldest quarter, precipitation seasonality, precipitation of driest quarter, and precipitation of warmest quarter. We conducted the analyses with default settings except random test percentage = 25 and replicates = 10. The mean value of the Area under the Receiver Operating Characteristic curve (AUC) was used to assess the model performance, with AUC > 0.9 regarded as reliable [52].

## Results

### Phylogeny

**Fig. 1:**
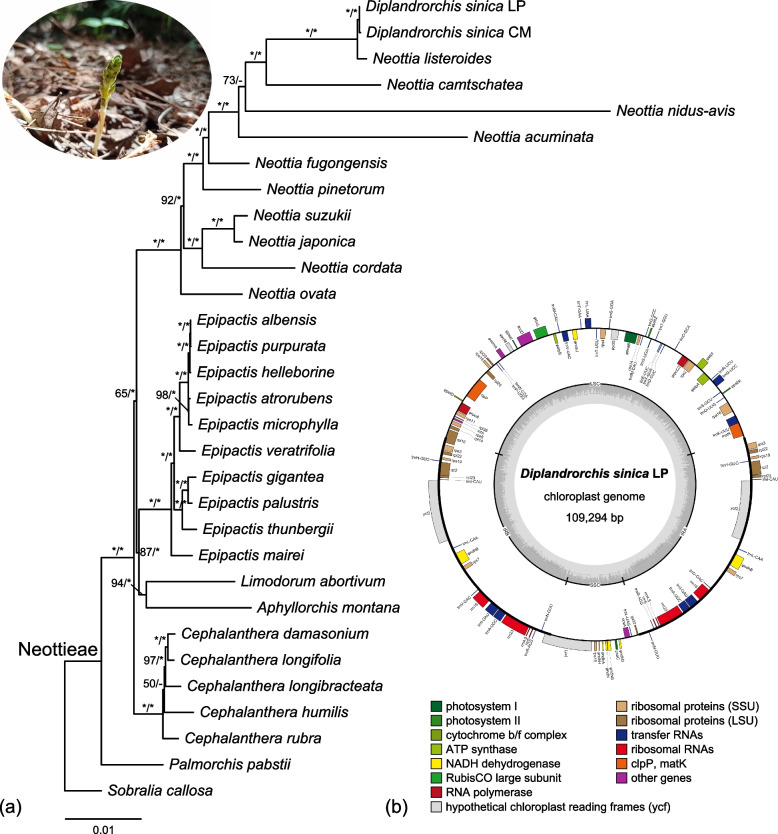
**a** Maximum likelihood tree of Neottieae inferred from the combined plastome and nuclear dataset. Numbers above branches are bootstrap values and Bayesian posterior probabilities. “*” indicate BS = 100% or PP = 1.0. “-” indicates the node not supported in the Bayesian analysis. **b** Circular gene map of the plastome of *Diplandrorchis sinica* from the Loess Plateau. Genes inside and outside the circle are transcribed in clockwise and counterclockwise directions, respectively. The dark gray inner circle corresponds to the G/C content, and the light gray circle represents the A/T content. Pseudogenes are marked with “φ”. Mycoheterotrophic *Diplandrorchis sinica* under the mixed deciduous broadleaf-conifer forest is presented in the upper left. Photographed by H.W. Peng. LP = Loess Plateau; CM = Changbai Mountains

Maximum likelihood and Bayesian analyses of the combined dataset yielded almost identical topologies with 89% nodes receiving strong support (BS ≥ 85% and PP = 1.0) (Fig. 1a). *Palmorchis* is the earliest-diverging lineage in Neottieae (BS = 100%, PP = 1.0), followed by *Cephalanthera* (BS = 65%, PP = 1.0). *Aphyllorchis* and *Limodorum* formed a clade (BS = 94%, PP = 1.0), sister to *Epipactis* (BS = 87%, PP = 1.0). *Diplandrorchis* was deeply embedded within *Neottia* and sister to *N. listeroides* (BS = 100%, PP = 1.0).

### Plastid gene loss and nutrition type reconstruction

**Fig. 2:**
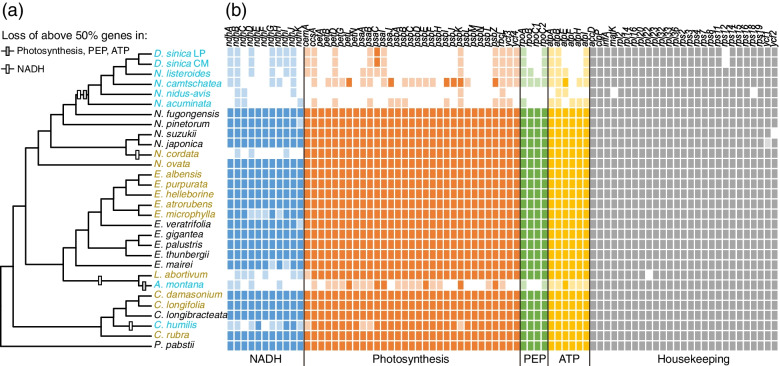
The evolution of plastomes in Neottieae. **a** Physically or functionally lost genes (> 50%) mapped on the ML tree. Mycoheterotrophic, mixotrophic, and autotrophic species are in blue, brown, and black, respectively. **b** Distribution patterns of protein-coding gene loss. The dark, light, and white boxes indicate presence, pseudogene, and absence of each gene, respectively. NADH = plastid NADH dehydrogenase-like complex; Phyotosynthesis = phyotosynthesis-related genes; PEP = plastid-encoded RNA polymerase; ATP = plastid ATP synthase

We reported the plastome of *D. sinica* for the first time. This plastome has a typical quadripartite structure, one large single copy (LSC), two inverted repeats (IR), and one small single copy (SSC) (Fig. 1b), but is highly reduced with a size of 109,294 bp, containing 49 protein-coding genes (including 22 putative pseudogenes), 30 transfer RNA genes, and 4 ribosomal RNA genes. The patterns of functional and physical loss of plastid protein-coding genes within Neottieae are mapped on the phylogeny (Fig. 2). Mixotrophic species are distributed in six clades belonging to four genera: *Cephalanthera*, *Epipactis*, *Limodorum*, and *Neottia*. There is no gene loss in mixotrophs of *Cephalanthera* and *Epipactis* except for *E. microphylla*, in which four *ndh* genes became pseudogenized. The plastome of *Limodorum* lost all *ndh*, *cemA*, and *rpl22* genes. In *Neottia*, the mixotrophic *N. ovata* did not lose any plastid gene, while *N. cordata* lost all *ndh* genes. For the sampled mycoheterotrophic species, *Cephalanthera humilis* lost the majority of *ndh* genes and four photosynthesis-related genes, while the other mycoheterotrophic species lost all *ndh* genes and the overwhelming majority of photosynthesis-related, *rpo* and *atp* genes. One (*rps12*) and two (*matK*, *rps18*) housekeeping genes were lost in *Diplandrorchis* and *N. nidus-avis*, respectively. In addition, loss of a few *ndh* or housekeeping genes occurred in some autotrophic species of *Epipactis* and *Neottia*.

**Fig. 3:**
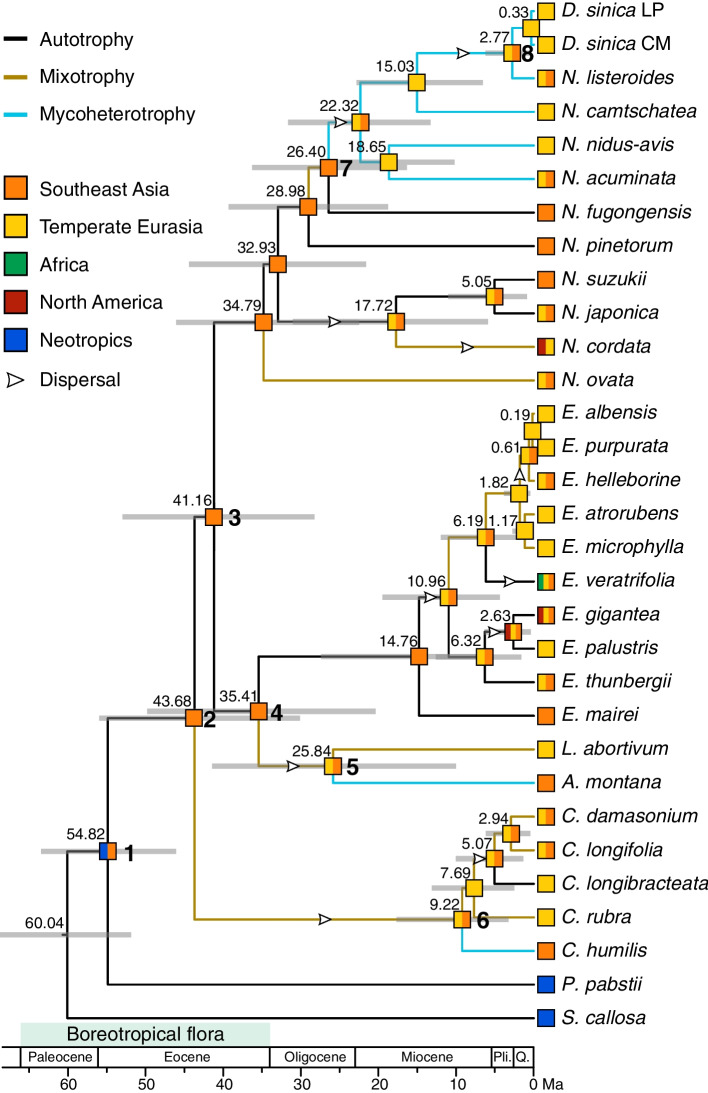
Evolutionary inference of nutrition types and ancestral range reconstruction for Neottieae. The chronogram with nodes represented by their mean ages was generated using BEAST. Gray bars represent 95% highest posterior density intervals. Nodes of interest are marked as 1–8 in bold. The most likely ancestral range estimated using BioGeoBEARS under the statistical DEC model is presented at each node as a colored square. The branch colors indicate ancestral nutrition strategy with the maximum probability inferred by BayesTraits. Pli. = Pliocene; Q. = Quaternary

Mapping of nutrition types on the phylogeny indicates that autotrophy is the ancestral state in Neottieae (Figs. 3 and S1). Mixotrophy has evolved independently at least five times. The autotrophic *C. longibracteata* originated from the mixotrophic ancestor. Mycoheterotrophic habit is distributed in three clades, *C. humilis*, *Aphyllorchis*, and a clade containing *Diplandrorchis* and four *Neottia* species, each of which is derived from the mixotrophic ancestor although in the last, one cannot exclude mycoheterotrophy to be derived from autotrophy directly (node 7 in Fig. S1).

### Divergence times and biogeography

Divergence time estimates and ancestral range reconstruction for Neottieae are shown in Figs. 3 and S2. The stem and crown group ages of Neottieae were estimated at 60.04 Ma (95% HPD: 51.85–68.70) and 54.82 Ma (95% HPD: 46.05–63.42; node 1), respectively. The most recent common ancestor of the tribe occupied Neotropics and Southeast Asia. The stem group ages of *Cephalanthera*, *Neottia*, and *Epipactis* were estimated at 43.68 Ma (95% HPD: 30.10–55.93; node 2), 41.16 Ma (95% HPD: 28.24–52.93; node 3) and 35.41 Ma (95% HPD: 20.37–49.75; node 4), respectively. All three mycoheterotrophic lineages originated from Southeast Asia. The split of *Aphyllorchis* and *Limodorum* occurred at 25.84 Ma (95% HPD: 10.00–41.39; node 5), *Cephalanthera humilis* diverged from other *Cephalanthera* at 9.22 Ma (95% HPD: 3.25–17.65; node 6), and the clade containing *Diplandrorchis* and four mycoheterotrophic *Neottia* species has diverged from its close relatives at 26.40 Ma (95% HPD: 16.35–36.24; node 7). *Diplandrorchis* originated at 2.77 Ma (95% HPD: 0.33–6.20; node 8) and the split of the Loess Plateau and Changbai Mountains populations was at 0.33 Ma (95% HPD: 0.003–1.01).

### Species distribution change of *D. sinica*

**Fig. 4:**
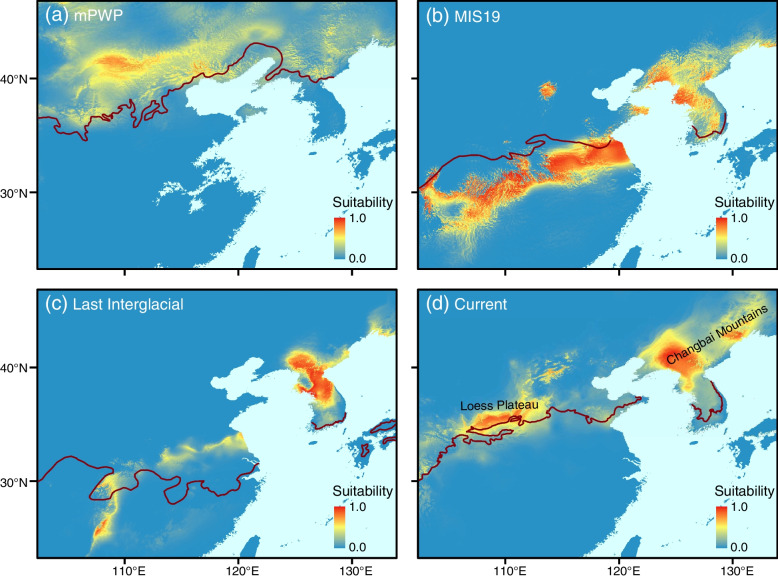
Potential distribution of *D. sinica* inferred by maxent modeling. **a** the mid-Pliocene Warm Period (mPWP; 3.205 Ma). **b** the Marine Isotope Stage 19 (MIS19; ~ 0.787 Ma). **c** the Last Interglacial (~ 0.13 Ma). **d** current conditions. The dark red lines indicate 0 °C isotherms derived from the mean temperature of coldest quarter in each period

The ENMs performed well at predicting the distribution patterns of *D. sinica* with the AUC value of 0.998. The most important predictive variable is the mean temperature of coldest quarter with 31.5% of the relative contributions, followed by the precipitation of warmest quarter (25%). Historical and current potential distributions of *D. sinica* are shown in Fig. 4. The projection of MaxEnt model suggests that *D. sinica* had a wide range near 40°N during the mPWP (3.205 Ma; Fig. 4a), and then migrated southward, which resulted in a discontinuous distribution between eastern China and the Korean Peninsula in the MIS19 (~ 0.787 Ma; Fig. 4b). In the Last Interglacial (~ 0.13 Ma), the distributional range largely shrank in these two regions (Fig. 4c). Under the current climatic condition, *D. sinica* migrated back to the north, largely corresponding to the present disjunct distribution between the Loess Plateau and Changbai Mountains (Fig. 4d).

## Discussion

### Systematic position of *Diplandrorchis* and the Loess Plateau-Changbai Mountains disjunction

Our phylogenetic analyses support *Palmorchis* as the earliest-diverging lineage in Neottieae, followed by *Cephalanthera*; *Aphyllorchis* is sister to *Limodorum* (Fig. 1a). These results are in agreement with previous studies [16, 17, 21]. *Diplandrorchis* was established by Chen [22], including *D. sinica*. Because of having two fertile stamens, Chen [22] regarded this genus as the most primitive in Neottieae. Govaerts [23] placed informally *Diplandrorchis* in *Neottia*, whereas Chen et al. [18] accepted it as a distinct genus. Our analyses indicate that *Diplandrorchis* was nested in *Neottia*. *Diplandrorchis*, *N. listeroides* and three other *Neottia* species (*N. acuminata*, *N. camtschatea*, and *N. nidus-avis*) formed a clade (BS = 100%, PP = 1.0). These five species are mycoheterotrophs, have a reduced plastome with extensive gene loss (Fig. 2), and are distributed in Eurasian temperate forests with *N. listeroides* and *N. acuminata* extending to East Asian subtropical forests [18, 39]. *Diplandrorchis sinica* is sister to *N. listeroides*, but they are obviously different in many morphological characters, such as non-resupinate flowers (vs. resupinate), modified lip absent (vs. present), two stamens (vs. one), and rostellum absent (vs. present) [18, 22]. *Neottia listeroides* is distributed in the Qinghai-Tibet Plateau and adjacent regions, while *D. sinica* is restricted to Changbai Mountains and Loess Plateau [18]. Thus, our data support the inclusion of *Diplandrorchis* in *Neottia*. Here we made the necessary taxonomic treatment.

***Neottia*** Guett., Hist. Acad. Roy. Sci. Mém. Math. Phys. (Paris, 4°) 1750: 374. 1754. TYPE: *N. nidus-avis* (L.) Richard.

= *Diplandrorchis* S.C.Chen, Acta Phytotax. Sin. 17(1): 2. 1979, syn. nov. TYPE: *Diplandrorchis sinica* S.C.Chen.

***Neottia sinica*** (S.C.Chen) Huan-Wen Peng & Wei Wang, **comb. nov.** ≡ *Diplandrorchis sinica* S.C.Chen in Acta Phytotax. Sin. 17(1): 2. 1979. TYPE: China, Liaoning, Huanren County, 25 Aug 1964, *S.C. Chui & J.C. Chu 245* (holotype, IFP-16408001z0001 [image!]; syntype, IFP-16408001z0002 [image!]).

*Neottia sinica* (= *D. sinica*) has a disjunct distribution between the Loess Plateau and Changbai Mountains. Our ENMs suggest the mean temperature of coldest quarter as the most powerful variable to predict potential distribution of *N. sinica*, which is sensitive to temperature changes and close to the 0 °C isotherms of the mean temperature of coldest quarter (Fig. 4). The molecular dating analysis suggests that *N. sinica* originated in the late Pliocene (ca. 2.77 Ma), during which this species might have had a relatively continuous distribution near 40°N in East Asia (Fig. 4a). From the latest Pliocene-Pleistocene, global temperatures decreased sharply [53] and East Asian winter monsoon intensified steadily [54], which resulted in the appearance of large and extensive glaciers in northern East Asia at ca. 0.9 Ma [55] and might thereby have driven *N. sinica* to migrate southward. The ENMs support the southward migration of this species during the Pleistocene, which resulted in its disjunct distribution between eastern China and the Korean Peninsula (Fig. 4b and c). The distributional range of *N. sinica* was contracted largely in these two regions in the Last Interglacial (Fig. 4c). Because the current temperature in East Asia, especially the mean temperature of coldest quarter, is higher than that of the Last Interglacial (Fig. 4; [51, 53]), *N. sinica* can slightly migrate back to the north (Fig. 4d). Thus, the Pleistocene climatic fluctuations and the resultant migration are responsible for the formation of the disjunction of *N. sinica* between the Loess Plateau and Changbai Mountains. Based on the average elevations, the Chinese mainland has been divided into three “steps” (high, middle and low) from the west to the east [56]. The Loess Plateau and Changbai Mountains belong to the second and third steps, respectively. This study provides the first case, to our knowledge, of an organismal group displaying the disjunction between the second and third steps, and therefore improves our understanding of evolution of East Asian biodiversity.

### Plastome degradation and origin of mycoheterotrophy in Neottieae

#### Shifts of nutrition types and plastid gene loss

Our evolutionary inference of nutrition types shows that autotrophy is the ancestral state in Neottieae (Fig. 3), in agreement with the result of Selosse and Roy [2]. Within Neottieae, at least five shifts from autotrophy to mixotrophy occurred, and mycoheterotrophy independently originated at least three times.

Among the five mixotrophic lineages in Neottieae, two (*Limodorum* and *N. cordata*) lost physically or functionally all eleven *ndh* genes. After the loss of *ndh* genes, the function of the NADH complex can be replaced by alternative nuclear-encoded genes [57, 58]. Thus, the mixotrophic species without the *ndh* genes can still retain photosynthesis ability, although the photosynthesis of *Limodorum* is inefficient [59]. The other three mixotrophic lineages did not lose any plastid gene except *E. microphylla* with four *ndh* pseudogenes (Fig. 2). For mixotrophs without plastid gene loss, such as *C. damasonium* and *C. rubra*, organic carbon from fungi might be used to supplement carbon by photosynthesis under low light conditions, and when exposed to suitable habitats, these species become almost autotrophic [60]. We found that the autotrophic *C. longibracteata* originated from the mixotrophic ancestor, and *C. longibracteata* and its mixotrophic allies do not lose any *ndh* gene. Thus, our analyses support the hypothesis that pseudogenization or physical deletion of the NADH complex is the first stage of plastid-encoded gene loss in the evolutionary breakdown of mycoheterotroph plastid genomes [11, 13]. The same loss or nonfunction of *ndh* genes were also found in many carnivorous plants, such as Lentibulariaceae [61] and Droseraceae [62]. Meanwhile, carnivorous *Nepenthes* × *ventrata* (Nepenthaceae) did not lose any *ndh* gene [63]. Carnivorous plants retain the photosynthetic function and can capture and digest small animals as a source of additional nitrogen and phosphorus, which allows them to survive in open habitats on nutrient-poor soils and in oligotrophic aquatic environments [64]. Parallel plastome degradations may therefore occur owing to the similar shift from autotrophy to heterotrophy [62, 63].

The loss of photosynthesis and purifying selection in photosynthesis-related genes result in rapid loss of such genes and plastome degradation in mycoheterotrophs (reviewed by [13]). In Neottieae, three shifts from mixotrophy to mycoheterotrophy occurred independently, and the extents of plastome degradation are different among the three mycoheterotrophic lineages (Fig. 2). *Cephalanthera humilis* lost eight of eleven of *ndh* genes and four photosynthesis-related genes, while its *rpo* and *atp* genes remain functional. This is conflicting with the viewpoint of Kim et al. [16] that photosynthesis-related, *atp* and *rpo* genes are lost simultaneously. *Aphyllorchis* lost all *ndh* and *rpo* genes and most photosynthesis-related and *atp* genes. In the mycoheterotrophic *Neottia* clade (including *Diplandrorchis*), the plastomes are highly reduced, especially in *N. sinica* and *N. nidus-avis*, a few housekeeping genes are also lost. We propose that plastome degradation in Neottieae is on the way and that the loss of photosynthesis-related genes is the second stage of plastome degradation, followed by *atp* and *rpo* gene loss [11, 14]. In *Aphyllorchis* and *N. camtschatea*, *atpE* is functional and other five *atp* genes are lost, whereas all *rpo* genes are lost, suggesting that the loss of *rpo* genes occurs earlier than that of *atp* genes, in agreement with the results of Graham et al. [13].

The shift from mycoheterotrophy back to mixotrophy/autotrophy was not found in our study. This means that mycoheterotrophy is an extreme end of mycorrhizal symbiosis [6, 65, 66], and that once lost, plastid genes could not be re-obtained [2]. In the evolutionary process of mycoheterotrophy, the loss of *ndh* genes first occurs, which can subsequently result in an irreversible evolutionary cascade of photosynthetic-related, ATP and PEP gene loss [13].

#### Evolutionary origin of mycoheterotrophy and the rise of local forests

Our molecular dating analysis suggests a stem group age of 60.04 Ma (95% HPD: 51.85–68.70) and a crown group age of 54.82 Ma (95% HPD: 46.05–63.42) for Neottieae, which are highly congruent with the estimates of Kim et al. [16] and Serna-Sánchez et al. [25]. Our molecular dating and ancestral range estimation analyses (Fig. 3) indicate the most recent common ancestor of Neottieae occurred in Southeast Asia and the Neotropics in the early Eocene (node 1), and the Old World major clades (nodes 2–4) diverged in Southeast Asia. The biogeographic study of the whole Orchidaceae suggests that this tribe originated from the present-day Neotropics [45]. In the early Cenozoic, a continuous and homogenous tropical flora occupied mid-latitudes across the Northern Hemisphere (“boreotropical flora”; [67]). Thus, boreotropical dispersal might be responsible for the early distribution of Neottieae.

Our evolutionary inference of nutrition types and ancestral range estimation show that the three mycoheterotrophic lineages and their sister groups are distributed in distinct temperature zones (Fig. 3). Irreversible photosynthesis gene loss prevents plants from regaining photosynthetic function and NADH system loss results in the inability to resist photo-oxidative stress, hampering plants from recolonizing high-light conditions [13, 68]. Accordingly, mycoheterotrophs usually inhabit understories of forests with low-light conditions. Increased canopy of forests contributes to the development of mycoheterotrophs [2]. Thus, we propose that range expansion, niche differentiation, and the rise of local forests might have driven the origins of mycoheterotrophs in Neottieae.

*Aphyllorchis* grows in tropical and southern subtropical Asian evergreen broad-leaved forests (EBLFs) [18], whereas its sister group (*Limodorum*) occurs in lowland to submontane oak, deciduous and chestnut forest in central and eastern Europe, North Africa and the Middle East [39]. Our molecular dating and ancestral range estimation analyses indicate that this genus originated from Southeast Asia at 25.84 Ma (95% HPD: 10.00–41.39; node 5), which temporally coincides with the intensification of the South Asian summer monsoon [69] and the establishment of the East Asian summer monsoon [70]. These two monsoon systems have influenced greatly Asian climate, which can bring high precipitation and consequently promote the development of EBLFs. Thus, the occurrence of the mycoheterotrophic *Aphyllorchis* may be in association with the development of Southeast Asian EBLFs in the late Oligocene.

*Cephalanthera humulis* inhabits subtropical EBLFs of northwestern Yunnan [71], while its sister clade originated in temperate deciduous forests [18, 39]. This mycoheterotrophic species originated at 9.22 Ma (95% HPD: 3.25–17.65; node 6), in line with the timing of the intensification of the East Asian summer monsoon [72, 73]. Molecular phylogenetics [74–76] and paleovegetation reconstruction [77, 78] have indicated that the rapid rise of East Asian subtropical EBLFs occurred during this period, which might thereby have driven the origin of subtropical East Asian mycoheterotrophs, such as *C. humulis*.

The mycoheterotrophic *Neottia* clade (including *Diplandrorchis*) is mainly restricted to the temperate Eurasian deciduous broad-leaved or coniferous forests with *N. listeroides* and *N. acuminata* extending to East Asian subtropical forests [18, 39], whereas its sister (*N. fugongensis*) grows in subtropical EBLFs on the eastern slope of the Gaoligong Mountains [79]. Our dating analysis indicate that this clade originated at 26.40 Ma (95% HPD: 16.35–36.24; node 7), in agreement with the timing of the “icehouse” state of the earth [80]. From the Oligocene onwards, microthermal broad-leaved deciduous and coniferous forests began to rise in mid-high latitudes [81–85] and thereby facilitated the origin of the temperate mycoheterotrophic *Neottia* clade.

## Conclusions

In this study, we present a well-resolved phylogeny for Neottieae based on plastome and nuclear ITS data. The Chinese *Diplandrorchis* was included in a molecular phylogenetic study for the first time and was recognized as a member of *Neottia*. Our molecular dating and ecological niche modeling analyses suggest that the Pleistocene climatic fluctuations and the resultant migration resulted in the disjunct distribution of *N. sinica* between the Loess Plateau and Changbai Mountains. In Neottieae, mycoheterotrophy might have evolved from mixotrophy with the staged and irreversible plastome degradation. This survival strategy is an extreme end of mycorrhizal symbiosis, which promoted mycoheterotrophs to adapt to forests with dense overstories and low light levels. Our findings suggest that mycoheterotrophic occurrence is in association with the rise of local forests. The Neottieae provides an empirical study to explain the evolutionary origins of mycoheterotrophs, as a result of local environmental changes.

## Supplementary Information

**Additional file 1: Table S1.** Taxa, distribution, and GenBank accession numbers for the sequences used in this study. **Table S2.** Comparison of the fit of different models of biogeographical range evolution and model-specific estimates for the different parameters. **Table S3.** Manual dispersal multipliers. **Table S4.** Georeferenced records of *D. sinica* used for species distribution modeling.

**Additional file 2: Fig. S1.** Evolutionary inference of nutrition types for Neottieae. Numbers in bold near branches indicate the node number, as referred to Fig. 3. Large pie charts show the relative probabilities of alternative ancestral states obtained by BayesTraits. **Fig. S2.** Ancestral range reconstruction for Neottieae using BioGeoBEARS under the statistical DEC model. Numbers in bold near branches indicate the node number, as referred to Fig. 3. Large pie charts show the relative probabilities of alternative ancestral distributions. Areas with probabilities below 5% are hidden and lumped together in black.

## Data Availability

All sequences in this study are available in the National Center for Biotechnology Information (NCBI) (https://www.ncbi.nlm.nih.gov/nuccore/), with GenBank accession numbers shown in Table S1.

## Abbreviations

AICc_wt: Weighted corrected Akaike Information Criterion
AUC: Area under the Receiver Operating Characteristic curve
BS: Bootstrap
ca.: Circa
CTAB: Cetyl trimethyl ammonium bromide
CM: Changbai Mountains
DEC: Dispersal–extinction–cladogenesis
DIVA: Dispersal–vicariance
EBLFs: Evergreen broad-leaved forests
ENM: Ecological niche modeling
HPD: Highest posterior density
IR: Inverted repeat
ITS: Internal transcribed spacer
LP: Loess Plateau
LSC: Large single copy
MCC: Maximum clade credibility
MCMC: Markov chain Monte Carlo
MIS: Marine Isotope Stage
ML: Maximum likelihood
mPWP: Mid-Pliocene Warm Period
Mt.: Mountain
PEP: Plastid-encoded RNA polymerase
PP: Posterior probability
SD: Standard deviation
SSC: Small single copy
