# Supplementary material for: Phylogenomics, plastome degradation and mycoheterotrophy evolution of Neottieae (Orchidaceae), with emphasis on the systematic position and Loess Plateau-Changbai Mountains disjunction of Diplandrorchis
Source: BMC Plant Biol. 2022 Nov 1;22:507. doi: 10.1186/s12870-022-03906-0 (PMC9624021; doi:10.1186/s12870-022-03906-0)
Supplement: Supplementary file 1 — Additional file 1: Table S1. Taxa, distribution, and GenBank accession numbers for the sequences used in this study. Table S2. Comparison of the fit of different models of biogeographical range evolution and model-specific estimates for the different parameters. Table S3. Manual dispersal multipliers. Table S4. Georeferenced records of D. sinica used for species distribution modeling. [file 12870_2022_3906_MOESM1_ESM.doc]

**Additional file 1**

**Table S1.** Taxa, distribution, and GenBank accession numbers for the sequences used in this study.

**Table S2.** Comparison of the fit of different models of biogeographical range evolution and model-specific estimates for the different parameters.

**Table S3.** Manual dispersal multipliers.

**Table S4.** Georeferenced records of *D. sinica* used for species distribution modeling.

**Table S1.** Taxa, distribution, and GenBank accession numbers for the sequences used in this study.

| Species | ITS | Plastome | Distribution |
| --- | --- | --- | --- |
| **Neottieae** |  |  |  |
| *Aphyllorchis montana* Rchb.f. | MZ463251 | NC_030703 | Tropical and Subtropical Asia |
| *Cephalanthera damasonium* (Miller) Druce | AY146446 | NC_041179 | Europe to Iran, Bhutan to SW. China |
| *Cephalanthera humilis* X.H.Jin | MZ463240 | NC_030706 | China (Yunnan) |
| *Cephalanthera longibracteata* Blume | AB856485 | NC_041180 | Russia (Far East) to NE. China, Japan |
| *Cephalanthera longifolia* (L.) Fritsch | AY146447 | NC_030704 | Europe to Mediterranean, China, Japan |
| *Cephalanthera rubra* (L.) Rich. | AY146445 | NC_041181 | Europe to Central Asia, NW. Africa |
| *Diplandrorchis sinica* S.C.Chen |  | MZ014629 | China (Liaoning) |
| *Diplandrorchis sinica* S.C.Chen | OP295487 | OP310037 | China (Gansu, Shaanxi) |
| *Epipactis albensis* Nováková & Rydlo | AY154384 | NC_041182 | S. Europe |
| *Epipactis atrorubens* (Hoffm.) Besser | JN847403 | NC_041183 | Europe to Caucasus |
| *Epipactis gigantea* Douglas | MF963894 | NC_041184 | SW. Canada to N. Mexico, China, India, S. Japan |
| *Epipactis helleborine* (L.) Crantz. | MZ463247 | NC_041185 | NW. Africa, Europe to China |
| *Epipactis mairei* Schltr. | MZ463250 | NC_030705 | Nepal to Central China |
| *Epipactis microphylla* (Ehrh.) Sw. | FR750399 | NC_041186 | S. Europe to Iran |
| *Epipactis palustris* (L.) Crantz | AY146448 | NC_041187 | Europe to Caucasus and Mongolia, China (Xinjiang) |
| *Epipactis purpurata* Sm. | FJ454871 | NC_041188 | Europe to N. Iran |
| *Epipactis thunbergii* A.Gray | KT338694 | NC_046817 | Russia (Far East) to S. Korea, Japan, China (Zhejiang) |
| *Epipactis veratrifolia* Boiss. & Hohen. |  | NC_030708 | Caucasus to Somalia and S. China |
| *Limodorum abortivum* (L.) Sw. | AY351378 | MH590355 | Europe to Mediterranean and N. Iraq |
| *Neottia acuminata* Schltr. | MZ463265 | NC_030709 | Himalaya to Temperate E. Asia |
| *Neottia camtschatea* (L.) Rchb.f. | KJ023677 | NC_030707 | Siberia to N. China |
| *Neottia cordata* (L.) Rich. | JN999304 | NC_041189 | Temperate and Subarctic Northern Hemisphere |
| *Neottia fugongensis* (X.H.Jin) J.M.H.Shaw | MZ463256 | NC_030711 | China (Yunnan) |
| *Neottia japonica* (Blume) Szlach. | MH321186 | NC_041446 | SE. China, Korea, Japan |
| *Neottia listeroides* Lindl. | MZ463262 | NC_030713 | Pakistan to Central China |
| *Neottia nidus-avis* (L.) Rich. | AY351383 | NC_016471 | Europe to Iran, NW. Africa |
| *Neottia ovata* (L.) Bluff & Fingerh. | FJ694841 | NC_030712 | Europe to Himalaya |
| *Neottia pinetorum* (Lindl.) Szlach. | MZ463259 | NC_030710 | Himalaya to subtropical China |
| *Neottia suzukii* (Masam.) Szlach. |  | NC_041447 | China (Taiwan) |
| *Palmorchis pabstii* Veyret |  | NC_041190 | French Guiana |
| **Outgroup** |  |  |  |
| *Sobralia callosa* | KT923833 | NC_028147 | Panama |

**Table S2.** Comparison of the fit of different models of biogeographical range evolution and model-specific estimates for the different parameters. Ln*L*, log-likelihood; *d*, rate of range expansion; *e*, rate of range contraction; *j*, relative per-event weight of jump dispersal; AICc, corrected Akaike Information Criterion; AICc_wt, weighted AICc. The best model with the highest AICc_wt vaule is in bold.

| Model | Ln*L* | Free parameter | | | | AICc | AICc_wt |
| --- | --- | --- | --- | --- | --- | --- | --- |
|  |  | Number | *d* | *e* | *j* |  |  |
| **DEC** | **-63.80** | **2** | **0.0002** | **1.00E-12** | **0** | **132.0** | **0.6** |
| DEC+*j* | -63.59 | 3 | 0.0002 | 1.00E-12 | 0.0029 | 134.1 | 0.22 |
| DIVA-like | -66.94 | 2 | 0.0003 | 1.70E-05 | 0 | 138.3 | 0.026 |
| DIVA-like+*j* | -66.94 | 3 | 0.0003 | 1.60E-05 | 0.0002 | 140.8 | 0.0076 |
| BayArea-like | -66.37 | 2 | 6.20E-05 | 0.0002 | 0 | 137.2 | 0.046 |
| BayArea-like+*j* | -64.28 | 3 | 4.60E-05 | 0.0002 | 0.0022 | 135.4 | 0.11 |

**Table S3.** Manual dispersal multipliers.

| Region | Southeast Asia | Temperate Eurasia | Africa | North America | Neotropics |
| --- | --- | --- | --- | --- | --- |
| **0–2 Ma** |  |  |  |  |  |
| Southeast Asia | 1 | 0.5 | 0.1 | 0.1 | 0.1 |
| Temperate Eurasia | 0.5 | 1 | 1 | 0.1 | 0.1 |
| Africa | 0.1 | 1 | 1 | 0.1 | 0.1 |
| North America | 0.1 | 0.1 | 0.1 | 1 | 1 |
| Neotropics | 0.1 | 0.1 | 0.1 | 1 | 1 |
| **2–30 Ma** |  |  |  |  |  |
| Southeast Asia | 1 | 0.5 | 0.1 | 0.1 | 0.1 |
| Temperate Eurasia | 0.5 | 1 | 1 | 0.1 | 0.1 |
| Africa | 0.1 | 1 | 1 | 0.1 | 0.1 |
| North America | 0.1 | 0.1 | 0.1 | 1 | 0.5 |
| Neotropics | 0.1 | 0.1 | 0.1 | 0.5 | 1 |
| **> 30 Ma** |  |  |  |  |  |
| Southeast Asia | 1 | 0.5 | 0.1 | 0.1 | 0.1 |
| Temperate Eurasia | 0.5 | 1 | 1 | 0.5 | 0.1 |
| Africa | 0.1 | 1 | 1 | 0.1 | 0.1 |
| North America | 0.1 | 0.5 | 0.1 | 1 | 0.5 |
| Neotropics | 0.1 | 0.1 | 0.1 | 0.5 | 1 |

**Table S4.** Georeferenced records of *D. sinica* used for species distribution modelling.

| No. | Locality | Longitude (°E) | Latitude (°N) | Source |
| --- | --- | --- | --- | --- |
| 1 | Liaoning, China | 124.9141944 | 41.34220833 | [1] |
| 2 | Liaoning, China | 124.9202778 | 41.34388889 | [2] |
| 3 | Liaoning, China | 124.8205541 | 41.32063521 | Chen YA 251 (IFP) |
| 4 | Liaoning, China | 124.830189 | 41.323811 | This study |
| 5 | Gansu, China | 108.5216806 | 35.65953889 | BNU, ZWL-9 (BNU) |
| 6 | Shaanxi, China | 108.6854444 | 35.65247222 | BNU, S13-07 (BNU) |
| 7 | Gansu, China | 108.528116 | 35.649198 | This study |

**References**

1. Zhang H, Zhang H, Fu J, He H, Zhu Y, Wang Y, Lan Y, Ma M. Habitat characteristics of endangered plant *Diplandrorchis sinica* population in Mt. Laotudingzi of eastern Liaoning province. J Liaoning Norm Univ (Nat Sci Ed.). 2014;37:389–395.
2. Lv R. The distribution of *Diplandrorchis sinica* S.C. Chen and the new discovery of phenological observation. J Heilongjiang Vocat Inst Ecol Eng. 2017;30:16–17.
