# Supplementary material for: Phylogenomics, plastome degradation and mycoheterotrophy evolution of Neottieae (Orchidaceae), with emphasis on the systematic position and Loess Plateau-Changbai Mountains disjunction of Diplandrorchis
Source: BMC Plant Biol. 2022 Nov 1;22:507. doi: 10.1186/s12870-022-03906-0 (PMC9624021; doi:10.1186/s12870-022-03906-0)
Supplement: Supplementary file 2 — Additional file 2: Fig. S1. Evolutionary inference of nutrition types for Neottieae. Numbers in bold near branches indicate the node number, as referred to Fig. 3. Large pie charts show the relative probabilities of alternative ancestral states obtained by BayesTraits. Fig. S2. Ancestral range reconstruction for Neottieae using BioGeoBEARS under the statistical DEC model. Numbers in bold near branches indicate the node number, as referred to Fig. 3. Large pie charts show the relative probabilities of alternative ancestral distributions. Areas with probabilities below 5% are hidden and lumped together in black. [file 12870_2022_3906_MOESM2_ESM.pdf]

Additional file 2

Fig. S1. Evolutionary inference of nutrition types for Neottieae. Numbers in bold near branches indicate the node number, as referred to Fig. 3. Large pie charts show the relative probabilities of alternative ancestral states obtained by BayesTraits.

Fig. S2. Ancestral range reconstruction for Neottieae using BioGeoBEARS under the statistical DEC model. Numbers in bold near branches indicate the node number, as referred to Fig. 3. Large pie charts show the relative probabilities of alternative ancestral distributions. Areas with probabilities below 5% are hidden and lumped together in black.

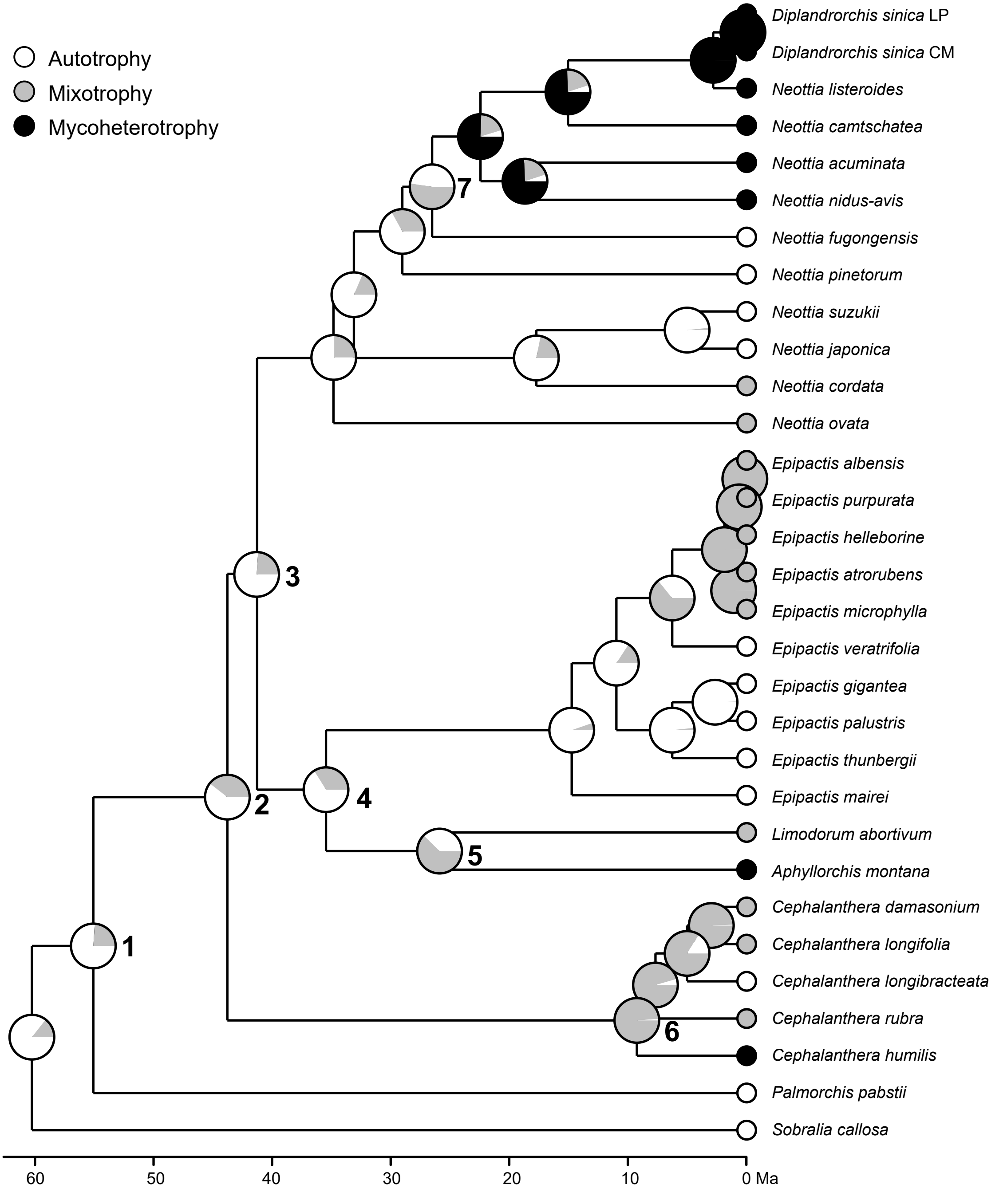
Autotrophy
Mixotrophy
Mycoheterotrophy
Diplandrorchis sinica LP
Diplandrorchis sinica CM
Neottia listeroides
Neottia camtschatea
Neottia acuminata
Neottia nidus-avis
Neottia fugongensis
Neottia pinetorum
Neottia suzukii
Neottia japonica
Neottia cordata
Neottia ovata
Epipactis albensis
Epipactis purpurata
Epipactis helleborine
Epipactis atrorubens
Epipactis microphylla
Epipactis veratrifolia
Epipactis gigantea
Epipactis palustris
Epipactis thunbergii
Epipactis mairei
Limodorum abortivum
Aphyllorchis montana
Cephalanthera damasonium
Cephalanthera longifolia
Cephalanthera longibracteata
Cephalanthera rubra
Cephalanthera humilis
Palmorchis pabstii
Sobralia callosa
1
2
3
4
5
6
7
60
50
40
30
20
10
0 Ma

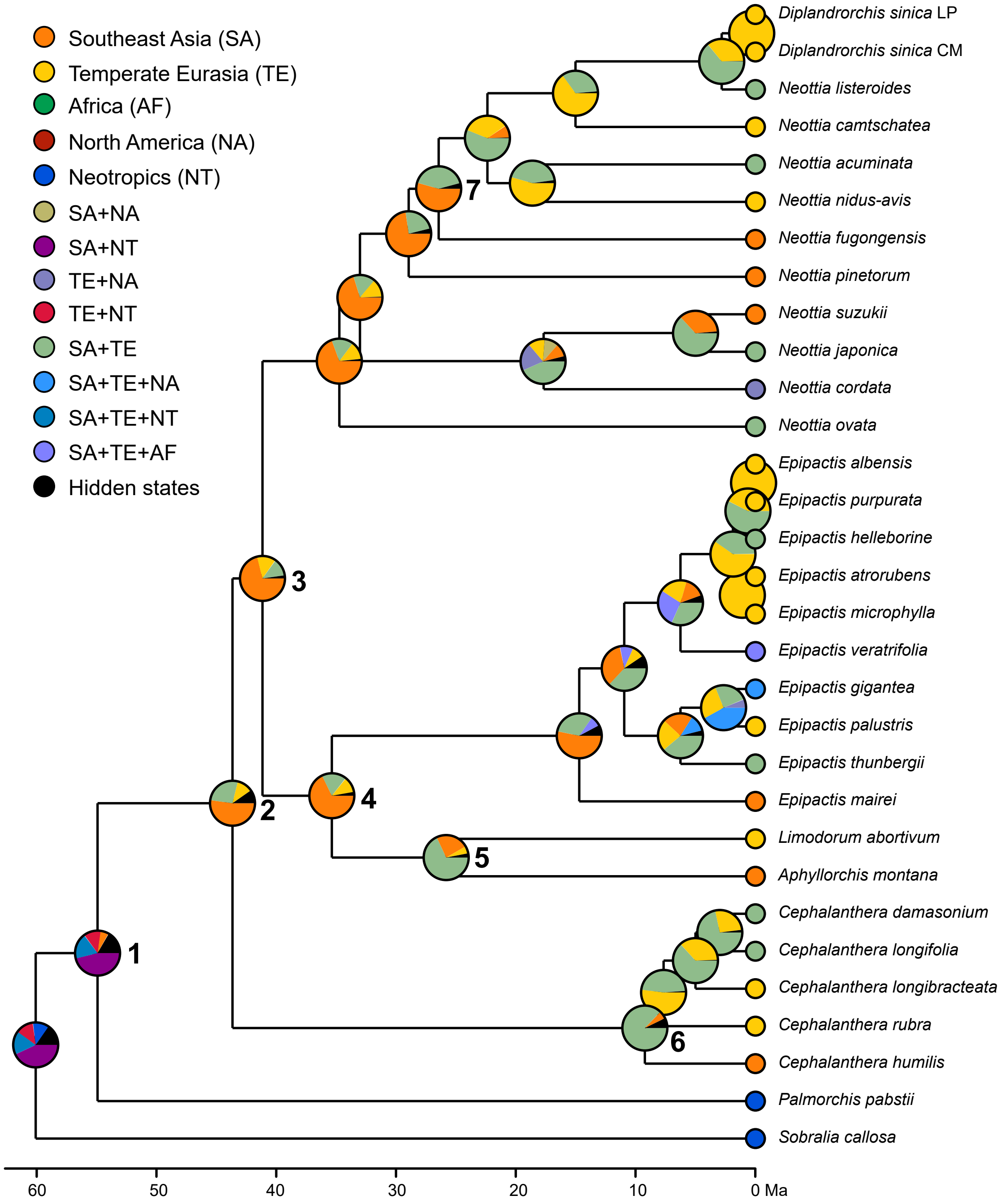
Southeast Asia (SA)
Temperate Eurasia (TE)
Africa (AF)
North America (NA)
Neotropics (NT)
SA+NA
SA+NT
TE+NA
TE+NT
SA+TE
SA+TE+NA
SA+TE+NT
SA+TE+AF
Hidden states
Diplandrorchis sinica LP
Diplandrorchis sinica CM
Neottia listeroides
Neottia camtschatea
Neottia acuminata
Neottia nidus-avis
Neottia fugongensis
Neottia pinetorum
Neottia suzukii
Neottia japonica
Neottia cordata
Neottia ovata
Epipactis albensis
Epipactis purpurata
Epipactis helleborine
Epipactis atrorubens
Epipactis microphylla
Epipactis veratrifolia
Epipactis gigantea
Epipactis palustris
Epipactis thunbergii
Epipactis mairei
Limodorum abortivum
Aphyllorchis montana
Cephalanthera damasonium
Cephalanthera longifolia
Cephalanthera longibracteata
Cephalanthera rubra
Cephalanthera humilis
Palmorchis pabstii
Sobralia callosa
1
2
3
4
5
6
7
60
50
40
30
20
10
0 Ma
